# Provider attributes correlation analysis to their referral frequency and awards

**DOI:** 10.1186/s12913-016-1338-1

**Published:** 2016-03-14

**Authors:** Matthew T. Wiley, Ryan L. Rivas, Vagelis Hristidis

**Affiliations:** Department of Computer Science and Engineering, University of California, Riverside, CA USA; SmartDocFinder LLC, 3499 10th Street, Riverside, CA USA

**Keywords:** Provider attributes, Provider referrals, Referral frequency correlation, Provider quality designation, Consumer health informatics, Patient decision making

## Abstract

**Background:**

There has been a recent growth in health provider search portals, where patients specify filters—such as specialty or insurance—and providers are ranked by patient ratings or other attributes. Previous work has identified attributes associated with a provider’s quality through user surveys. Other work supports that intuitive quality-indicating attributes are associated with a provider’s quality.

**Methods:**

We adopt a data-driven approach to study how quality indicators of providers are associated with a rich set of attributes including medical school, graduation year, procedures, fellowships, patient reviews, location, and technology usage. In this work, we only consider providers as individuals (e.g., general practitioners) and not organizations (e.g., hospitals). As quality indicators, we consider the referral frequency of a provider and a peer-nominated quality designation. We combined data from the Centers for Medicare and Medicaid Services (CMS) and several provider rating web sites to perform our analysis.

**Results:**

Our data-driven analysis identified several attributes that correlate with and discriminate against referral volume and peer-nominated awards. In particular, our results consistently demonstrate that these attributes vary by locality and that the frequency of an attribute is more important than its value (e.g.*,* the number of patient reviews or hospital affiliations are more important than the average review rating or the ranking of the hospital affiliations, respectively). We demonstrate that it is possible to build accurate classifiers for referral frequency and quality designation, with accuracies over 85 %.

**Conclusions:**

Our findings show that a one-size-fits-all approach to ranking providers is inadequate and that provider search portals should calibrate their ranking function based on location and specialty. Further, traditional filters of provider search portals should be reconsidered, and patients should be aware of existing pitfalls with these filters and educated on local factors that affect quality. These findings enable provider search portals to empower patients and to “load balance” patients between younger and older providers.

**Electronic supplementary material:**

The online version of this article (doi:10.1186/s12913-016-1338-1) contains supplementary material, which is available to authorized users.

## Background

Recently, there has been an increased interest in provider search portals such as Vitals.com and Healthgrades.com [1, 2]. A key challenge for these portals is to identify attributes that determine the quality of a provider, and to make these attributes available to their users. Provider search portals typically allow users to rank providers by location, patient rating, or last name, and users may filter providers by medical school or affiliated hospital rankings. However, ranking based on patient reviews may be ineffective as the wide majority of patient ratings are positive, and previous research has shown that patients mostly rate providers on office wait times and visit durations [3–7]. Further, better medical schools do not necessarily create better providers, as a provider’s residency has a stronger impact on that provider’s clinical style [8].

Other studies have assessed the qualitative attributes of provider quality via surveys [9–12]. These studies show that accurate diagnosis and treatment, probity, good communication and listening skills, sensitivity towards feelings, and tailoring treatment options are the qualitative attributes of provider quality. Unfortunately, measuring these qualitative attributes for all providers is impossible given the available information on providers and provider search portals. CMS may publish performance data for individual providers in the future, such as medical procedure outcomes, but more subjective attributes such as listening skills may still be largely unavailable.

Given the lack of data on qualitative attributes and the sparsity and bias of patient reviews of provider quality, we focus on quantitative attributes of providers in this study. There is a rich set of data available for each provider, however, a key challenge in using a data-driven approach is finding the ground truth–i.e., a set of “good” providers–to guide our analysis of important attributes for provider quality. The Centers for Medicare and Medicaid Services (CMS) has defined quality measures, such as the Physician Quality Reporting System (PQRS), but PQRS data is only publicly available for group practices with more than 25 providers and hence is not applicable to individuals [13].

In our approach we view referral frequency and peer-nominated quality designations as indicators for provider quality, although we understand that these measures have their own flaws and limitations as discussed in the limitations section. We view both peer-nominated awards and referral frequency as a peer-validated quality measures—i.e., a provider would not receive many referrals or nominations if he or she has not garnered the trust of their peers, which implies high-quality ratings from the local community. We adopt a data-driven approach to discover the provider attributes that are associated with these quality indicators. Our focus is to study the correlations among a wide range of provider attributes and indicators of quality, keeping in mind that correlation is not equal to causation, nor are our quality measures comprehensive (unfortunately there are no comprehensive quality indicators for individual providers that are publicly available).

### Related work

The related work can be split into two categories: provider search sites and attributes associated with provider quality. Previous work shows that providers are being rated online, as one out of every six physicians has been rated online [14]. Moreover, provider rating websites have observed increases in usage from less than 1 % to over 30 % for specific specialties from 2005 to 2010 [14]. Further, several studies have attempted to identify attributes of provider quality, but these studies focus on qualitative aspects of medical practice (e.g., communication skills) rather than quantitative aspects (e.g., medical school rank).

### Online provider search sites

There has been increased interest in provider search portals with over 30 studies and reviews appearing in peer-reviewed journals [15, 16]. The previous related work has studied the topic of provider ratings online, but these studies are focused solely on user generated content and do not consider the rich set of provider data readily available. Ellimoottil et al. studied online reviews of 500 urologists from Vitals.com and found that each physician was rated 2.4 times on average and 86 % of physicians had positive ratings [4]. Wan and Dimov analyzed online reviews of 300 allergists from three popular provider review websites, and they also found that a majority of reviews were positive [17]. Further, they reported a statistical difference when categorizing reviews by the physician’s graduation year, which showed that physicians who graduated more recently obtained more positive scores. Kadry et al. analyzed 4999 online provider ratings from the 10 most popular websites that rate providers, and they found that a majority of reviews are positive. Further, Kadry et al. suggest that a single overall rating to evaluate providers is sufficient to assess a patient’s opinion of a provider [5].

Verhoef et al. published a review on provider rating websites as tools to understand quality of care, and they found that several studies indicate a relationship between ratings and quality of care [15]. However, Verhoef et al. point out that provider rating websites have some drawbacks, including anonymity of ratings and the fact that the population on social media is not representative of the actual patient population. Due to the anonymity of the ratings, the overall scores of each provider are susceptible to fraud [15]. Hence, provider ratings may not be reliable for assessing the quality of a provider. Segal et al. examined online surgeon reviews and whether those reviews are able to track surgeon volume [18]. They showed that high volume surgeons can be differentiated from lower volume surgeons by using the number of ratings, the number of text comments for a surgeon, and the ratio of positive and negative comments.

### Attributes associated with provider quality

Several surveys have examined the qualitative attributes of providers and, but none have focused on the quantitative attributes of providers. Lee et al. assessed the attributes that make a good provider by generating a list of characteristics and surveying medical students, faculty, patients, and primary care providers [9]. Their survey showed that all participants regarded accurate diagnosis and treatment as the most important attribute and keeping up-to-date as the second most important attribute. Lambe and Bristow also surveyed a panel of experts from a wide range of medical specialties on the most important attributes of good providers [10]. They found that probity, recognition that patient care is the primary concern of a provider, good communication and listening skills, and recognition of one’s own limits were among the top attributes. As with Lee et al., Labe and Bristow sought to identify qualitative attributes of top providers.

Schattner et al. surveyed 445 patients at hospitals and clinics, asking each patient to select the four most important attributes from a questionnaire of 21 arbitrary attributes [12]. The most essential attributes selected were professional expertise, patience and attentiveness, informing the patient, and representing the patient’s interest. Further, Schattner et al. found that significantly more attributes were selected in the domain of patient’s autonomy over the domain of professional expertise. Luthya et al. also examined attributes of good providers from the patient’s perspective via a survey [11]. They found that sensitivity towards feelings and tailoring treatment options were the most important attributes for good providers. Similar to the other studies, Luthya et al. focused on the qualitative attributes of good providers.

None of the aforementioned studies—on both provider search sites and attributes of provider quality—have performed a data-driven, quantitative analysis of provider attributes. Hence, research is lacking on the association between information from provider rating websites and publicly available data, such as the patient’s perspective via user reviews, credentials of the provider (e.g., medical school), and professional attributes (e.g., accepted insurance plans). This leaves several data-driven questions unanswered. E.g., which attributes determine a peer-nominated award, and do these attributes also correlate with attributes that determine a provider’s referral frequency? And, are reviews based on wait times useful for finding distinguished providers, or providers who receive many referrals?

## Methods

**Fig. 1:**
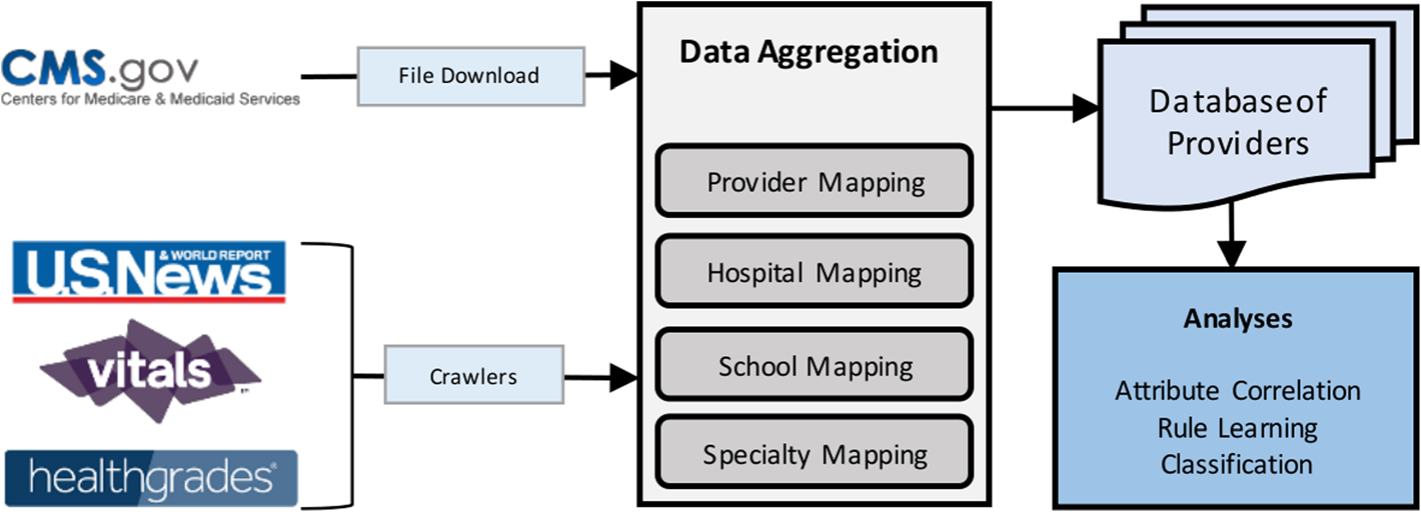
An overview of our methods from data collection to aggregation to analysis

We collected detailed data from a diverse set of sources including CMS data on providers and hospitals, U.S. News rankings of medical schools and hospitals, and additional provider information and patient reviews from Vitals.com and Healthgrades.com. We then mapped entities across sources, creating a database of 608,935 providers; this database is then used in each of our analyses. We converted each provider’s information to a set of intuitive quantitative attributes. For instance, medical school, residency, and fellowship were converted to integers based on the U.S. News & World Report (“U.S. News”) medical school rankings [19–21]. Affiliated hospitals were mapped to specialty-specific rankings as defined by U.S. News (e.g., cancer, gynecology, urology, etc.). Figure 1 presents an overview of our methods.

### Quality indicators

For referrals we selected CMS’s 2012–2013 30 day interval public dataset of Medicare and Medicaid referral patterns [22]. In this data set, referrals are only considered when a provider services a patient 30 days after another provider serviced the same patient—given that the first provider is listed as a referring provider on the second provider’s CMS claim. Medicare Part A and B beneficiaries, in most cases, do not need referrals to see specialists enrolled in Medicare; however Medicare Part C beneficiaries on Healthcare Maintenance Organization (HMO) plans are required to have a referral to see a specialist (certain exceptions exist, such as annual mammogram screenings) [23, 24]. In 2013, 9.3 of the 50 million Medicare beneficiaries were enrolled in a Part C HMO plan, up from 8.5 million in 2012; in both years these beneficiaries accounted for 65 % of all Part C beneficiaries [25, 26]. Thus approximately 20 % of all Medicare beneficiaries must obtain a referral to see a specialist; moreover, regardless of insurance plan, most radiological procedures require a physician referral. Further, primary care physician referrals are amongst the leading factors patients consider when choosing physicians [27].

For rule learning and classification purposes, Referral Frequency is converted into a nominal attribute with five distinct values based on the provider’s referral frequency relative to other providers:

1. None (never referred, e.g., a general practitioner)
2. Very Low (normalized referrals greater than 0 and less than or equal to 0.25)
3. Low (normalized referrals greater than 0.25 and less than or equal to 0.5)
4. High (normalized referrals greater than 0.5, less than or equal to 0.75)
5. Very High (normalized referrals greater than 0.75).

For quality designation we selected the Castle Connolly designation; each year Castle Connolly distinguishes top providers both nationally and regionally through a peer nomination process that involves over 50,000 providers and hospital and healthcare executives [28]. Castle Connolly receives over 100,000 nominations each year, and a physician-led research team awards top providers from these nominations. Regional awardees are leaders in their communities and national awardees are physicians who attract patients from across the country [29]. Analogous to Castle Connolly, several organizations have internal peer-nominated awards (e.g., Kaiser Permanente Medical Group awards; the American Academy of Family Physicians awards Family Physician of the Year). However, unlike Castle Connolly, these types of awards are not as comprehensive nor do they consider a wide pool of physicians across several specialties. Hence we focus on Castle Connolly awards as other awards are limited by the number of awardees and their geographical and medical specialty diversity.

### Data collection

Insurance information and patient ratings were collected from both Vitals.com and Healthgrades.com [1, 2]. Medical school and hospital rankings were collected from U.S. News’s reports [19, 21]. CMS has released several datasets for health providers (and hospitals) based in the U.S. This includes general information such as the provider’s specialties, medical training, and hospital affiliations [30, 31]. Other provider information includes the Healthcare Common Procedure Coding System (HCPCS), physician referrals, and prescription data [22, 32, 33]. Note that all CMS datasets link providers using a National Provider Identifier (NPI). CMS hospital information includes name, location, and a unique identifier which is used to link each NPI to affiliated hospitals [34]. CMS data was downloaded directly from cms.gov [22, 30–34]. Separate crawlers were built using jsoup [35]—a Java library that obtains and parses HTML pages—for each of the other data sources: Vitals.com, Healthgrades.com, and U.S. News.

In total, we collected information on 3.2 million distinct providers from CMS, 4600 distinct hospitals from CMS, 1.9 million distinct providers from Healthgrades.com, 1 million distinct providers from Vitals.com, 1,956 hospitals from U.S. News, and 149 distinct medical schools from U.S. News. After appropriate data transformations and entity mappings, we generated the set of provider attributes listed in Tables 1 and 2.

**Table 1:** List of attributes used in our analysis based on the data collected (continued in Table 2)

Category	Attribute	Description	Source
Quality Indicators	Referral Frequency	Normalized number of referrals.	CMS
	Castle Connolly Award	Whether or not the provider is recognized by Castle Connolly as a distinguished provider.	Vitals.com
General Information	Gender	Male or female, as specified in the CMS data.	CMS
	Accepting New Patients	Whether or not the provider is accepting new patients.	Vitals.com and Healthgrades.com
	Specialties	A set of attributes, one for each specialty, e.g., cardiologist.	CMS
	Census Division	One of the nine regional divisions as defined by the U.S. Census Bureau, based on the provider’s location [36].	CMS
	Number of Organization Members	Number of organization members, e.g., 1 for a private practice with 1 provider.	CMS
	Languages	A set of attributes that represent languages spoken by the provider.	Healthgrades.com
	Number of Spoken Languages	The number of spoken languages spoken by the provider.	Healthgrades.com
	Accepts Medicare Insurance	Whether or not the provider accepts Medicare assignments.	CMS
	PQRS	Whether or not the provider participates in the Physician Quality Reporting System (PQRS)[13].	CMS
	EHR	Whether or not the provider uses an Electronic Health Record (EHR) system.	CMS
	eRx	Whether or not the provider uses electronic prescriptions.	CMS

**Table 2:** List of attributes used in our analysis based on the data collected (continued from Table 1)

Category	Attribute	Description	Source
HCPCS Information	Procedure Types	A set of binary attributes, one for each type of procedure performed by the provider. HCPCS cover anything billable to Medicare, from new visits to transplants.	CMS
	Relative Cost of Procedures	The relative cost of the provider’s procedures, normalized to [0,1] by all providers within a 30 mile radius.	CMS
	Relative Procedure Volume	The relative volume of the provider’s procedures, normalized to [0,1].	CMS
	Number of HCPCS Beneficiaries	Number of beneficiaries for all HCPCSs for the provider.	CMS
Prescriber Information	Prescription Types	The types of drugs prescribed by the provider (brand and generic names handled separately).	CMS
	Number of Rx Beneficiaries	Number of Medicare beneficiaries from the prescriber dataset.	CMS
Hospital Affiliations	Affiliated Hospital Score	The maximum score from the provider’s hospital affiliations, where the score of each hospital affiliation depends upon the provider’s specialties and U.S. News scoring of hospitals.	CMS (to get hospitals) and U.S. News (for score of hospitals)
	Number of Affiliated Hospitals	Number of hospital affiliations for the provider.	
Insurance	Number of Accepted Insurances	Number of insurers accepted by the provider.	Vitals.com and Healthgrades.com
	Individual Insurers	A set of attributes, one for each insurer accepted by the provider, e.g., Humana.	Vitals.com and Healthgrades.com
Medical Experience	Medical School Rank	Ranking of the provider’s medical school by primary care rating.	CMS and U.S. News
	Years of Experience	The difference between 2014 and the year the provider graduated medical school.	CMS
	Credentials	The provider’s credentials, e.g., MD, DO, FACP, etc.	CMS
	Residency Rank	Ranking of the provider’s residencies by primary care rating.	Healthgrades.com and U.S. News
	Fellowship Rank	Ranking of provider’s fellowships by primary care rating.	Healthgrades.com and U.S. News
	Number of Residencies	Number of the provider’s residencies.	Healthgrades.com
	Number of Fellowships	Number of the provider’s fellowships.	Healthgrades.com
Disciplinary Information	Number of Malpractices	Number of malpractices of the provider.	Healthgrades.com
	Number of Sanctions	Number of sanctions of the provider.	Healthgrades.com
	Number of Board Actions	Number of disciplinary board actions of the provider.	Healthgrades.com
Average Ratings from Patient Reviews	Patient Review Ratings	A set of attributes based on user reviews: Overall Rating, Ease of Appointment, Follows Up After Visit, Promptness, Spends Time with Me, Courteous Staff, Bedside Manner, and Accurate Diagnosis.	Merge reviews from Vitals.com and Healthgrades.com
	Number of Patient Reviews	Number of patient reviews for the provider.	Vitals.com and Healthgrades.com

The Referral Frequency attribute is log transformed as its distribution is observed to be exponential; we then normalize Referral Frequency to the interval [0,1]. Analogous transformations are applied to the Relative Cost of Procedures and Relative Procedure Volume attributes. Years of Experience and all variables with the prefix “Number” are represented as numeric attributes. A few of the attributes are single binary variables, such as electronic prescriptions (eRx) and Accepting New Patients. Attributes that appear as combinations are represented as sets of binary attributes, including Credentials, Specialties, Languages, Procedure Types, Prescription Types, and Individual Insurers. Methods for computing values for Medical School Rank, Residency Rank, Fellowship Rank, and Affiliated Hospitals’ Score are described in the next subsection.

### Entity mappings

The names of medical schools and hospitals listed by U.S. News differ from the names in the CMS data. E.g., “University of California, Riverside,” “University of California — Riverside” and “UC Riverside” all refer to the same school. Therefore, we used a string edit distance metric—the minimum number of operations (insert and delete) to transform one string into another string—to map CMS names to U.S. News names for all medical schools and hospitals with more than 100 occurrences; each of these mappings were then manually reviewed as some results were incorrect or no mappings exist (as in cases where a medical school is located outside of the U.S. or a hospital is not listed by U.S. News). This generated 231 medical school mappings and 2029 hospital mappings. The medical school mappings were then used to assign values for each provider’s Medical School Rank*,* Fellowship Rank, and Residency Rank, where null (unknown value) is used for providers whose medical schools are missing from the mappings.

The hospital rankings listed by U.S. News scores hospitals across several specialties for adults and children; for each hospital listed, the hospital’s score, name, location, and rankings were collected. Further, the hospital specialties reported by U.S. News do not always correspond to the specialties listed by CMS. In particular, CMS uses a taxonomy of medical specialties that consider subspecialties whereas U.S. News uses broad categories for specialties [37]. Note that this mapping is not necessarily one-to-one; e.g., a provider specializing in internal medicine may map to several categories listed by U.S. News. Therefore, we manually mapped all specialties with more than 100 occurrences to the specialties used by U.S. News. CMS specialties are self-selected by providers; 195 of the 653 specialties have less than 100 providers. These rare specialties included technicians (e.g., Biomedical Engineering), therapists (e.g., Poetry Therapist), Clinical Nurse Specialists (a majority of nurses are marked as practitioners instead of specialists), and Molecular Genetics. This generated 5651 mappings. We then used these mappings to assign scores to each of the affiliated hospitals. For each affiliated hospital, we compute the average score of the hospital with respect to the provider’s specialties as a hospital’s score varies by specialty. We then assign Hospital Affiliation Score to the hospital affiliation with the maximum score (i.e., the best affiliation), where null values are used for providers whose hospital affiliations are missing from the hospital mappings.

Several attributes were collected from our crawlers, including Castle Connolly Award, Accepting New Patients, language, fellowship, residency, disciplinary actions, and patient reviews information. Thus for each provider, we mapped their CMS data to Vitals.com and Healthgrades.com provider profiles. In particular, we mapped 608,935 providers between CMS, Vitals.com, and Healthgrades.com; 25,514 of whom have received a Castle Connolly award. To map CMS providers to providers from other sources, we followed a hybrid automatic-manual data integration approach. First, we identified a promising set of attributes to use for mapping, specifically: first name, middle name, last name, address, medical school, graduation year, affiliated hospitals, and specialties. For each attribute we constructed a customized mapping algorithm. For example, the mapping between first names is computed using the Levenshtein distance between the two strings; medical schools and hospitals used their respective mappings. Then, we assigned weights to each attribute’s matching score based on a large number of accuracy experiments, where the authors defined the ground truth mappings. We then computed a mapping threshold based on the mapping scores via more accuracy experiments. We obtained a precision of 100 % and a recall of 94 % for our Vitals.com mapping, and a precision of 98 % and a recall of 93 % for our Healthgrades.com mapping.

### Attributes analysis and classification methods

We examined the information gain and correlation of each of the attributes from Tables 1 and 2 with respect to Castle Connolly Award and Referral Frequency. Information gain is used to filter the set of attributes such that only discriminative attributes are correlated and employed for classification. We then mined rules using RIPPER, a rule learning algorithm, and classified Castle Connolly Award and Referral Frequency to validate the selected attributes [38]. Rule learning algorithms (e.g.*,* RIPPER) are employed to discover relationships between attributes in large data sets; for example, given a dataset of transactions at a supermarket, a rule learning algorithm discovers which items are commonly bought together. Weka, an open source set of tools for data mining, was employed in each of our analyses [39].

As expected, we found that the data is highly imbalanced for both Castle Connolly Award and Referral Frequency. Only 4 % of all mapped providers have received a Castle Connolly award and 42 % of all mapped providers have zero referrals; a majority of providers with zero referrals specialized in Internal Medicine, Family Medicine or Emergency Medicine. This imbalance poses computational challenges for rule learning and hinders trivial classifiers. Further, only analyzing the data at the national level will omit local trends, such as state-wide Electronic Health Record (EHR) and eRx incentive programs. Thus we stratified our original dataset by each provider’s state and perform our rule learning and classification tasks at both the national and state levels. Intuitively, attributes that may be discriminative in California are not the same attributes that are discriminative in New York. Moreover, healthcare is regulated both at the state and federal levels. These regulations, along with demographics and population health, create localized trends in healthcare.

We investigated the classification task using random forests and 5-fold cross-validation. Random forests has been shown to work well on imbalanced datasets [40, 41]. We applied cost-sensitive training to each classifier, where each example is weighted based on its output label. Thus, the model treats errors from each class label equally. For example, given 100 training examples with two classes, an even split would have 50 positive examples and 50 negative examples; however, if only 4 examples are positive, then applying a weight of 50/4 = 12.5 for each positive example, and a weight of 50/96 = 0.52 for each negative example will yield a cost-sensitive dataset where both the positive and negative examples are treated equally. Further, cost-sensitive training allows each classifier to make meaningful classifications; otherwise a classifier could simply guess false for Castle Connolly Award and obtain a precision of 96 % and a sensitivity of 0 %.

Each experiment used a 5-fold cross validation for training and testing purposes. In all experiments we set the number of trees to 20, the maximum depth to 1 + (0.01 * n) and number of features to 1 + (0.025 * n), where n is the number of features. These parameters, which are modeled after the default parameters, were chosen using a validation phase, where we enumerated different combinations of all three parameters and validated the settings on three randomly selected states; we repeated the random selection of states ten times for each combination. As noted in the methods, we used cost-sensitive training datasets, that weigh each example based on its class label, to avoid trivial classifiers (e.g., always classifying Castle Connolly Award = false yields a classifier with 96 % accuracy).

## Results

In this section we report the results of our analyses for Referral Frequency = Very High and Castle Connolly Award = true. First we report some general statistics on Castle Connolly Award = true and Referral Frequency = Very High. Next we report correlations between Referral Frequency and Castle Connolly Award, along with correlations of attributes. Last, we present a summary of our classification results. Detailed rule learning results are reported in Additional file 1: Appendix E.

### General statistics of providers

**Fig. 2:**
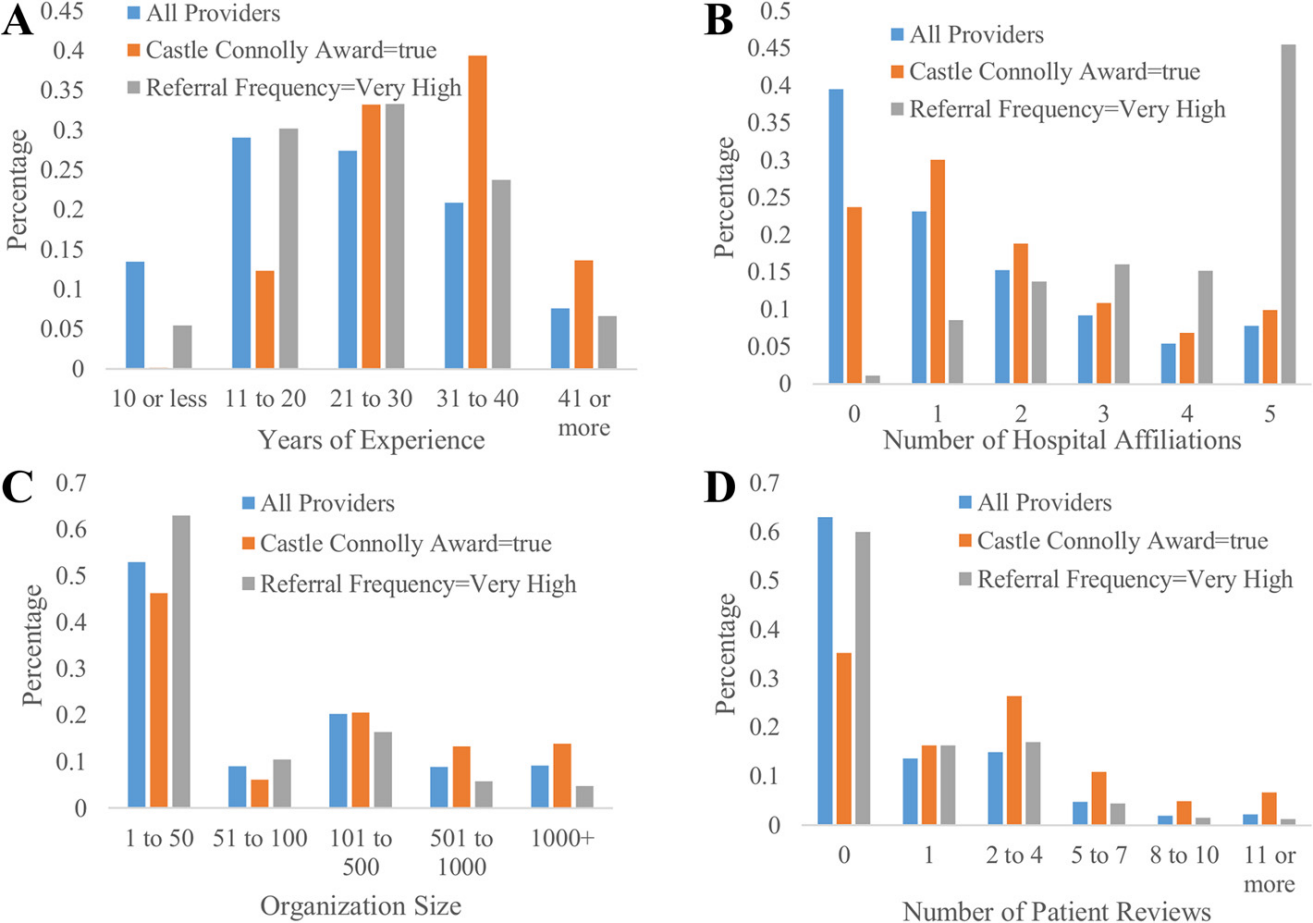
Distributions of YearsExp, NumHospitals, NumOrgMembers, and NumReviews for all providers, Castle Connolly Award = true, and Referral Frequency = Very High

First we analyzed some general statistics and demographics of providers at the national level; demographics of providers are presented in Additional file 1: Appendix A. Figure 2(a-d) presents the distributions of Years of Experience, Number of Affiliated Hospitals, Number of Organization Members, and Number of Patient Reviews for all providers, Castle Connolly Award = true, and Referral Frequency = Very High. Several interesting observations may be made from Fig. 2. Firstly, providers that receive many referrals are likely to have at least a decade of experience or they are likely to be affiliated with several hospitals; however, patient review frequency and organization size have less of an impact on referral frequency. On the other hand, a provider is more likely to receive a Castle Connolly award if she or he has over 10 years of experience, works at a larger organization, and receives at least 1 or more reviews online. Assuming the average age of a student entering medical school is 22, that medical school requires four years of training, a majority of providers with a Castle Connolly award are between the ages 46 and 66.

Table 3 lists the top 10 specialties ranked by the proportion of providers who have Referral Frequency = Very High; Wilcoxon signed-rank tests showed all differences to be significant with p less than 0.001. As expected, radiology and its subspecialties have a high concentration of providers who are referred frequently. Interventional cardiology and internal medicine is the only top 10 specialty not related to radiology; this is likely because heart disease is the leading cause of death for both men and women in the U.S. [42]. Further, interventional cardiology and internal medicine accounts for over 23 % of providers with Referral Frequency = Very High.

**Table 3:** Top 10 specialties ranked by the proportion of providers who have Referral Frequency = Very High

Specialty	Total Number of Providers for Given Specialty	Percentage of Referral Frequency = Very High within Given Specialty
Diagnostic Ultrasound	760	52.1 %
Body Imaging	1076	51.9 %
Neuroradiology	1725	50.7 %
Diagnostic Neuroimaging	169	49.1 %
Vascular and Interventional Radiology	1558	48.8 %
Diagnostic Radiology	15,957	48.1 %
Nuclear Radiology	1011	47.5 %
Pediatric Radiology	629	45.3 %
Nuclear Cardiology	7991	47.5 %
Interventional Cardiology and Internal Medicine	3817	40.5 %

Table 4 lists the top 10 specialties ranked by the proportion of Castle Connolly awards within the respective specialty; Wilcoxon signed-rank tests showed all differences to be significant with p less than 0.001. Pediatric and oncology specialists have higher rates of Castle Connolly awards than general specialties, such as internal medicine with a rate of 2 % or family medicine with a rate of 1 %. However, internal medicine has the highest number of Castle Connolly awards, accounting for 9.8 % of all Castle Connolly awards.

**Table 4:** Top 10 specialties ranked by the proportion of Castle Connolly awards within the respective specialty

Specialty	Total Number of Providers for Given Specialty	Percentage of Castle Connolly Award = true within Given Specialty
Gynecologic Oncology	980	29.6 %
Pediatric Surgery	926	24.8 %
Reproductive Endocrinology	429	12 %
Pediatric Urology	97	23.7 %
Oncology Surgery	1021	22.3 %
Pediatric Nephrology	550	21.8 %
Otology and Neurotology	205	20.9 %
Colon and Rectal Surgery	1445	20.0 %
Pediatric Pulmonology	1081	18.8 %
Pediatric Endocrinology	1058	18.8 %

### Attribute correlations and discriminative power

We computed the correlation of Referral Frequency and Castle Connolly Award = true, along with the average number of referrals for Castle Connolly Awards. We found that the Pearson correlation of Referral Frequency and Castle Connolly Award is positive, but very low, specifically 0.058. However, this low correlation is not surprising as Castle Connolly Award reflects peer recognition whereas Referral Frequency reflect patient volume. Further, a provider with high volume may not necessarily be recognized as an outstanding provider, or an outstanding provider may not necessarily have high volume. For example, a provider may receive a referral because he or she is prompt to perform a test and has an efficient office, and not necessarily because he or she is an outstanding provider. Hence, high referrals and peer awards can be viewed as just two of the possible quality indicators, describing different quality aspects.

Table 5 reports strong and negligible correlations of attributes with respect to referral frequency. Several of these correlations are due to the nature of referrals, thus we focus on nonobvious correlations. Unexpected correlations include:

**Table 5:** Selected correlations of attributes with respect to referral frequency. The p-value for all correlations is less than 0.01, except for the ones with an asterisk

Strong Correlations	Correlation
HCPCS: Initial Hospital Care	0.46
Number of Hospital Affiliations	0.43
Number of HCPCS Beneficiaries	0.33
Relative Procedure Volume	0.26
HCPCS: X-ray Exam of Abdomen	0.24
Number of Rx Beneficiaries	0.23
Internal Medicine and Cardiovascular Disease	0.22
Diagnostic Radiology	0.21
Family Medicine	−0.20
Obstetrics and Gynecology	−0.16
Number of Fellowships	0.10
Negligible Correlations	
Hospital Score	−0.02
Patient Review Ratings	[−0.01,0.01]^*^
Number of Patient Reviews	−0.01
Number of Accepted Insurances	0.02
Years of Experience	0.01
Medical School Rank	0.03
Individual Insurers	[−0.4,0.5]^*^
Residency Rank	0.01^*^

1. User ratings and number of reviews are negligibly correlated with referral frequency. Hence, referrals are more likely based on physician-to-physician trust, and establishing relationships with other physicians could be more important than being popular with patients.
2. Referral Frequency is strongly correlated with the number of affiliated hospitals and the total number of affiliations is more important than the score of the respective affiliations.
3. Years of experience and insurance information are negligibly correlated with referral frequency. That is, simply accepting more insurance plans or practicing medicine for a longer period of time is not sufficient to secure more referrals.

We also examined correlations of Referral Frequency = Very High at the state level with the aim to observe local trends in providers with frequent referrals, as reported in Additional file 1: Appendix B.

A majority of attributes have negligible correlations (less than or equal to 0.05) with respect to Castle Connolly Award = true, except for those attributes listed in Table 6. This table suggests that providers with Castle Connolly awards have a diverse set of attributes; however, providers that see new patients or speak multiple languages are more likely to have a Castle Connolly award. We report state-level correlations of Castle Connolly Award = true in Additional file 1: Appendix B, which, among other results, reports a correlation for female gender in nine states.

**Table 6:** Attributes with a correlation greater than 0.05 with respect to Castle Connolly Award = true. The p-value for all correlations is less than 0.001

Attributes with correlation greater than 0.05	Correlation
HCPCS: New Office/Outpatient Visit	0.13
Language = Spanish	0.08
Insurance = Aetna Health	0.06
Number of Spoken Languages	0.06

Table 7 reports the top 10 most discriminative attributes for Castle Connolly Award in terms of information gain. This table suggests that whether a provider has a Castle Connolly award may be discriminated by the quantity of an attribute rather than the value of the attribute*.* E.g.*,* the number of patient reviews of a provider is more discriminative than the review scores; the number of fellowships and residencies is more discriminative than the institution rankings. The top 10 most discriminative attributes for Referral Frequency are reported in Additional file 1: Appendix C.

**Table 7:** The top 10 most discriminative attributes for Castle Connolly Award in terms of information gain

Most Discriminative Attributes for Castle Connolly Award
Number of Fellowships
Years of Experience
Number of Patient Reviews
HCPCS: New Office/Outpatient Visit
Number of Residencies
Accepting New Patients
Number of Organization Members
Number of Accepted Insurances
Family Medicine
Number of Spoken Languages

### Classification results

We evaluated classifiers at the national level and state level using the parameters from the methods for both Referral Frequency and Castle Connolly Award. In both cases, state-by-state classifiers outperformed national classifiers; state-level results are reported in Additional file 1: Appendix D. Thus, finding discriminative attributes to classify Castle Connolly providers or providers with high referral frequency is easier using attributes at the local level, and these local influencers should be modeled in each classifier separately.

Table 8 reports the confusion matrix for the discretized Referral Frequency classifier at the national level. For Referral Frequency = Very High, we observed an accuracy of 96 %, sensitivity of 52 %, specificity of 98 %, and a positive predictive value of 78 %. A majority of errors (Type I and Type II) were either classified as or labeled as Referral Frequency = High. Errors for other categories were similar, where a majority of errors occurred relative to the ordering of categories; compare Referral Frequency = Low with Referral Frequency = Very Low and Referral Frequency = High. Thus, provider referral frequency may be discretized and classified at the national level, with reasonable accuracies due to the correlations of attributes with referrals frequency.

**Table 8:** Confusion matrix of discretized Referral Frequency at the national level

Classified as →	Referral Frequency = None	Referral Frequency = Very Low	Referral Frequency = Low	Referral Frequency = High	Referral Frequency = Very High
Referral Frequency = None	225,329	22,576	4652	3603	462
Referral Frequency = Very Low	7289	22,637	11,136	504	1
Referral Frequency = Low	5540	18,936	60,688	16,107	26
Referral Frequency = High	2187	2347	31,522	131,589	4916
Referral Frequency = Very High	219	92	350	16,789	19,260

Table 9 reports the confusion matrix for the Castle Connolly classifier at the national level. Based on this table we observed a balanced sensitivity, specificity, accuracy, and precision, 77 %. However due to the large number of false negatives, our positive predictive value is not as promising at 13 %; although a trivial classifier would have a positive predictive value of 0 %. Hence peer awards are difficult to predict based on the attributes of a provider. State-level classifiers observed more accurate results, as reported in Additional file 1: Appendix D.

**Table 9:** Confusion matrix of Castle Connolly Award at the national level

Classified as →	Castle Connolly Award = false	Castle Connolly Award = true
Castle Connolly Award = false	448,689	130,927
Castle Connolly Award = true	5791	19,623

## Discussion

Our results have demonstrated and identified several attributes that are both correlated and discriminative for providers who are frequently referred. Further, we showed that most correlations are negligible with Castle Connolly awards at the national level, which suggests that a one-size-fits-all approach to ranking providers is inadequate. However, we demonstrated that these attributes are indeed discriminative for both referral frequency and Castle Connolly awards via rule learning and classification, and that these attributes are better discriminators at the state level due to local influencers. Hence, provider search portals should not use a global ranking formula across the whole country or across all specialties*,* but instead learn different weights for each attribute based on the user’s location or provider’s specialty.

Moreover, our findings have consistently demonstrated that the frequency of an attribute is more important than the value of an attribute—e.g.*,* the number of reviews of a provider is more important than the individual review ratings. Thus, current filters for provider search portals, such as medical school ranking, patient review rating, or hospital affiliation ranking, do not necessarily determine quality. Instead, emphasis should be placed on the number of reviews, fellowships, residencies, insurers, or hospital affiliations. The implication of these results is that quality of care is affected by providers who have a more diverse set of experiences and access to a larger set of services. Expanding services and increasing experience can be achieved through accepting more insurance plans and increasing hospital affiliations. Income is directly tied to rates of mortality, morbidity, and access to healthcare; thus accepting a wider range of insurance plans will expose the provider to a more diverse set of patients and episodes [43]. Further, hospital affiliations usually require an existing relationship—where leadership alignment promotes the collaboration. Thus, best practices are shared, along with an expansion of services in a cost-effective manner [44]. Lastly, providers who encourage patients to author reviews will have a more comprehensive picture of their skills online, even if they are a 3 or 4-star doctor. As the 5-star doctor with a handful of reviews may have solicited these reviews from family and friends, and thus the 5-star rating is inaccurate.

Further, the locality of quality factors should also be captured when ranking providers, as pointed out in the Appendix. For example, states with higher rates of Castle Connolly awards suggest more nominations, and hence more providers seek peer-review processes such as accreditation programs, which have been shown as tools to increase quality of care [45]. Similarly, demographics and credentials affect referral rates and Castle Connolly awards. E.g., nine states report correlations between females and Castle Connolly awards whereas zero states report correlations for males, and 50 of 51 states (including Washington D.C.) have correlations with pediatricians. Moreover, our rule learning results show that factors such as specific prescriptions or procedures affect referral frequency dependent upon locality, and varying years of experience and organization size affect Castle Connolly awards dependent upon locality.

Hence patients should be educated on the local factors that determine provider quality within their community, and patients should be made aware of the pitfalls of existing filters in provider search portals. For example, patients should compare the number of hospital affiliations of each provider with the average number of hospital affiliations of providers in the patient’s community, and patients should be aware that a majority of patient reviews are scored based on wait times and visit durations. This education would allow provider search portals to highlight younger providers with less years of experience who have attributes in common with older providers who have high marks in quality. Hence our work enables provider search portals to empower patients and to “load balance” patients between younger and older providers without sacrificing quality of care.

The next stage of this research will include more performance measures and patient survey data as they are made available by CMS and other sources. We expect performance measures to correlate with quality, and hence these measures should improve the accuracy of our inferences and predictions. We also plan to integrate organizational attributes into our algorithms, such as payment data and performance measures of hospitals. For example, CMS has released surveys of patients’ experience with hospitals, which reports hospital-level attributes such as doctor and nurse communication, cleanliness of hospital environment, and willingness to recommend the hospital [46]. Integrating organizational data and performance measures will enable us to build a provider reputation rating system, where, for each provider, we identify attributes that would improve the provider’s reputation.

### Limitations

A limitation of this work is that our results are tied to CMS, Vitals.com, and Healthgrades.com data. This analysis depends on successfully mapping between these data sources, and the accuracy of these data sources is not guaranteed; e.g., errors made by an optical character recognition program—a popular method for amassing data from PDF files—will create inaccurate data. Moreover, attributes change over time. Consider a provider who moves to a new office and updates his or her address with CMS, but Vitals.com has yet to process the update. Thus, these two sources become inconsistent and mappings are unsuccessful as location is a critical factor when mapping providers. Other attributes that become inconsistent over time include: last name, subspecialties, and hospital affiliations. Further, providers who do not participate in Medicare and Medicaid will have several missing attributes, and referrals outside of Medicare and Medicaid are omitted. However, we collected data on and successfully mapped 608,935 providers. Another limitation is that a majority of providers have zero reviews; this is likely due to the fact that only 4 % of Internet users post online reviews for providers, and previous work has shown that most providers have zero reviews [14].

Another limitation is the usage of referral frequency and Castle Connolly awards as quality indicators. Firstly, these indicators are not comprehensive—CMS has defined measures for physician quality via PQRS, but this data is currently not publicly available at the provider level. Further, PQRS measures are condition specific, and while this information is useful for a provider search portal, our analysis focused on a condition insensitive analysis of provider quality. We understand that the number of referrals greatly depends on the specialty; normalizing this number by the specialty could potential lead to another quality measure. Further, while the Castle Connolly award is prestigious and rigorously vetted, the award is biased towards providers who have more experience, because providers with more experience have had more time to build their reputation. However, our results show that several other attributes are also discriminative and years of experience alone does not determine a Castle Connolly designation.

## Conclusions

We studied which attributes from a provider’s profile correlate with and discriminate against referral volume and peer-nominated awards. Our findings have shown that a one-size-fits-all approach to provider ranking is inadequate, and that local influencers on provider quality must be considered when ranking providers. In turn, patients should be aware of the pitfalls of current provider search portals, and patients should be educated on the local factors influencing provider quality. Provider search portals that integrate these findings effectively will empower patients and enables these portals to “load balance” patients between younger and older providers without sacrificing quality of care.

## Additional file

Additional file 1: Appendix of provider demographics, state-level analyses, and rule learningresults [47]. (PDF 234 kb)

## Abbreviations

CMS: Centers for Medicare and Medicaid Services
PQRS: Physician Quality Reporting System
HMO: Healthcare Maintenance Organization
NPI: National Provider Identifier
EHR: Electronic Health Records
eRx: Electronic Prescriptions
