# Supplementary material for: Provider attributes correlation analysis to their referral frequency and awards
Source: BMC Health Serv Res. 2016 Mar 14;16:90. doi: 10.1186/s12913-016-1338-1 (PMC4790057; doi:10.1186/s12913-016-1338-1)
Supplement: Additional file 1: — Appendix of provider demographics, state-level analyses, and rule learningresults [47]. (PDF 234 kb) [file 12913_2016_1338_MOESM1_ESM.pdf]

Appendix A Demographics of Providers

Table A.1 reports the distributions of single binary attributes for Castle Connolly Award=true and Referral Frequency=Very High for all providers; Wilcoxon signed-rank tests showed all differences to be significant with p less than 0.001. This table contains some interesting observations, in particular, providers who receive many referrals or a Castle Connolly award are more likely to accept new patients and Medicare patients; further, these providers also more likely to participate in PQRS, EHR, and eRx systems. The gender result is less surprising; according to a 2012 census of active physicians 70% of doctors are male and 30% are female [1].

Table A.1 Distribution of single binary attributes.

	Percentage among providers with Castle Connolly Award=true (25,514 providers)	Percentage among providers with Referral Frequency=Very High (36,712 providers)	Percentage among All Providers
Gender=Male	79.6%	86.2%	69.2%
Accepting New Patients=true	84.0%	72.7%	55.5%
Accepts Medicare Insurance=true	71.1%	81.3%	56.7%
PQRS=true	34.7%	50.1%	24.6%
EHR=true	20.0%	23.9%	11.8%
eRx=true	32.8%	43.2%	21.5%

We visualized the ratio of providers with Referral Frequency=Very High and Castle Connolly Award=true over the total number of providers for each state using a heat map, shown in Figures A.2 and A.3. As shown in Figure A.2, Nevada and the mid and south Atlantic regions of the U.S. have the highest concentration of providers with Referral Frequency=Very High, which may imply that a majority of referral services are concentrated to a smaller number of providers in these areas due to a lack of specialists. As shown in Figure A.3, the north east region of the U.S. contains a higher concentration of providers with Castle Connolly awards than any other region in the U.S. Further, Florida, Washington, and Indiana also contain a considerably high ratio of Castle Connolly awards (greater than 5%). These results suggest that more providers in these states seek peer validation, which may result in a greater number of medical or clinical peer reviews. And peer review processes, such as accreditation programs, are tools to improve provider quality-of-care [2].

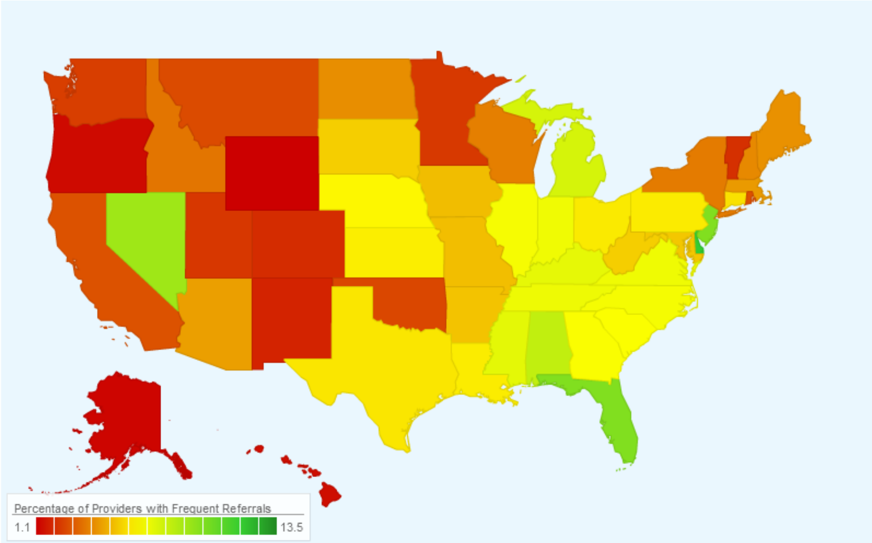

Figure A.2 Ratio of providers with Referral Frequency=Very High to the total number of providers by state. This map was generated using the Google Visualization API and used according to terms described in the Creative Commons 3.0 Attribution License [3,4].

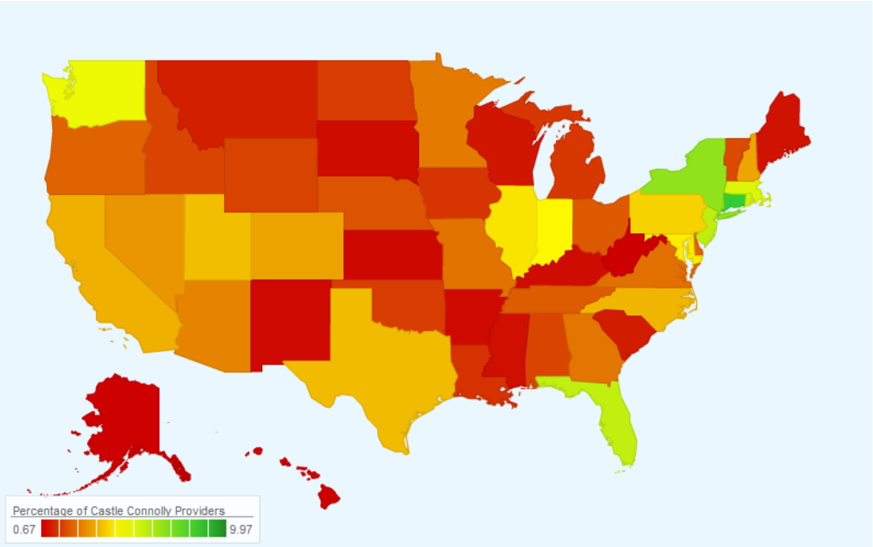

Figure A.3 Ratio of providers with Castle Connolly Award=true to the total number of providers by state. This map was generated using the Google Visualization API and used according to terms described in the Creative Commons 3.0 Attribution License [3,4].

Appendix B State-Level Correlations

Here we present our analysis of state-level correlations with Referral Frequency=Very High in order to observe local trends in providers with frequent referrals. We found that 75 distinct attributes have a correlation greater than 0.05 when the data is stratified by each state. A majority of these attributes had correlations greater than 0.05 in one or two states; Table B.1 lists the top 10 most frequently correlated attributes at the state level (note that the total number is 51 as Washington D.C. is included). Based on this table, there is indeed local influences on providers who are frequently referred, and these influencers are dominated by pediatric specialties.

Table B.1. The top 10 most frequently correlated attributes for Referral Frequency=Very High at the state level.

Attribute	Number of States
Pediatrics	50
Accepts Medicare Insurance	49
Emergency Medicine	49
Neonatal-Prenatal Medicine	48
Psychiatry	47
Child and Adolescent Psychiatry	45
Pediatric Critical Care Medicine	37
Pediatric Hematology-Oncology	37
Pediatric Cardiology	34
Obstertrics and Gynecology	33

We also examined correlations of Castle Connolly Award=true at the state level and found that 82 distinct attributes have a correlation greater than 0.05 when the data is stratified by each state. A majority of these attributes had correlations greater than 0.05 in one or two states; Table B.2 lists the top 10 most frequently correlated attributes at the state level. Based on this table, Castle Connolly awards indeed observe localized behavior and this behavior is influenced by the provider's specialty. This localized behavior could be explained by the peer-nomination process employed by Castle Connolly. Further, we also see local trends for certain types of drugs, such as Metformin for Type II diabetes and Cyclobenzaprine for muscle spasms. Lastly, despite the overrepresentation of males in Castle Connfolly (79% versus 69% overall), we see that female has a correlation greater than 0.05 with Castle Connolly Award=true in nine states whereas male had zero states with a correlation greater than 0.05.

Table B.2. The top 10 most frequently correlated attributes for Castle Connolly Award=true at the state level.

Attribute	Number of States
Family Medicine	32
Internal Medicine	21
Emergency Medicine	18
Anesthesiology	17
HCPCS: Emergency Department Visit	11
Accepts Medicare Insurance	10
Prescription: Metformin HCL	9
Gender=Female	9
Prescription: Cyclobenzaprine HCL	6
Prescription: Azithromycin	6

Appendix C Most Discriminative Attributes for Referrals

To gain insight into attributes useful for classifying providers' referral frequency, we examined the top 10 most discriminative attributes for the discretized Referral Frequency attribute in Table C.1. This table shows that a provider's referral frequency may be discriminated by vascular-related prescriptions (e.g., Warfarin), if the provider offers electronic prescriptions, the provider's relative volume, if the provider is seeing new patients, and if the provider participates in PQRS. Note, the top three discriminative attributes from this table are also strongly correlated with Referral Frequency=Very High.

Table C.1 The top 10 most discriminative attributes for discretized Referral Frequency in terms of information gain.

**Most Discriminative Attributes for *Referral Frequency=Very High***
Number of HCPCS Beneficiaries
NumHospitals
HCPCS: Initial Hospital Care
HCPCS: New Office/Outpatient Visit
PQRS
eRx
Relative Procedure Volume
Prescription: Furosemide
Prescription: Warfarin
Prescription: Plavix

Appendix D Detailed Classification Results

Table D.1 reports the confusion matrix for the discretized Referral Frequency classifiers at the state level where each cell is tallied across all states. As with the national level, we see a majority of errors are relative to the ordering of categories. Further, we observe a significant improvement in sensitivity from 52% to 72% for Referral Frequency=Very High classifications, however there is no change in accuracy and some degradation in positive predictive value, from 78% to 70%. Other categories observed similar behavior except for Referral Frequency=Low, which observed a decrease in sensitivity. Thus, finding discriminative attributes to classify providers with high referral frequency is easier using attributes at the local level, and these local influencers should be modeled in each classifier separately. However, local influencers have less of an effect on classifying providers with very low referral frequency or no referrals.

Table D.1 Confusion matrix of Referrals at the state level. Each cell is tallied across all states.

Classified as →	Referral Frequency= None	Referral Frequency= Very Low	Referral Frequency= Low	Referral Frequency= High	Referral Frequency= Very High
Referral Frequency= None	232,331	13,972	6008	3453	858
Referral Frequency= Very Low	9134	17,417	14,293	720	3
Referral Frequency= Low	5781	11,172	64,310	19,896	137
Referral Frequency= High	1777	996	23,866	135,998	9923
Referral Frequency= Very High	112	12	210	9892	26,484

Table D.2 reports the confusion matrix for the Castle Connolly Award classifiers at the state level where each cell is tallied across all states. Compared to the national classifier, we observed a degradation in sensitivity but an improvement in accuracy, specificity, and positive predictive value with 88%, 89%, and 18% respectively. Further, states with a high concentration of Castle Connolly awards had higher positive predictive values, namely New York, Florida and Connecticut all had positive predictive values over 30%. Thus, finding discriminative attributes to classify Castle Connolly providers is easier using attributes at the local level, and these local influencers should be modeled in each classifier separately.

Table D.2 Confusion matrix of Castle Connolly Award at the state level. Each cell is tallied across all states.

Classified as →	Castle Connolly Award=false	Castle Connolly Award=true
Castle Connolly Award=false	518,986	64,372
Castle Connolly Award=true	11,385	14,023

Appendix E Rule Learning Results

In this section we report a summary of the rules found using the RIPPER algorithm on Castle Connolly Award and discretized Referral Frequency. For each dataset at the national and state level, we ran RIPPER with pruning, a maximum error rate of 50%, and the minimum number of items covered by a rule to 10; i.e., every rule evaluates to at least 10 positives and each rule has at most half the number of negatives. For every rule, at both the state and national levels, we computed its accuracy using the number of positives and negatives that the rule covers and present the rules that yield the highest accuracies; in the case of Referral Frequency, we only

report rules that cover at least 100 providers as there are several rules that cover more than 100 providers with 90% or better accuracy. Essentially, each rule is identifying a cadre of providers with similar qualities who either have a high referral frequency or received a Castle Connolly award. This qualitative analysis gives further insight into local influencers of highly referred providers and providers with a Castle Connolly award.

Table E.1 reports the top five most accurate rules that cover at least 100 providers for Referral Frequency=Very High. Based on the rules from this table, we indeed see that Number of Affiliated Hospitals and Number of HCPCS Beneficiaries are important factors in determining providers with Referral Frequency=Very High, but surprisingly, these rules do not consider specialties. Instead, every rule has an emphasis on Number of HCPCS Beneficiaries and four of the five rules contain Prescription: Hydrocodone-Acetaminophen=false. Thus—in addition to the number of hospital affiliations, and Medicare procedures and patients— providers who are highly referred perform specific laboratory procedures that differ based on locality and these same providers tend to avoid a specific medication unique to the locality.

Table E.1. The top five most accurate features for rules that imply Referral Frequency=Very High.

State	Rule	Positive	Negative	Accuracy
PA	Number of HCPCS Beneficiaries >= 2855 AND Number of Organization Members >= 13 AND Prescription: Hydrocodone-Acetaminophen=false AND Prescription: Avapro=false AND HCPCS: Electrocardiogram Report= true	154	1	99.3%
NC	Number of HCPCS Beneficiaries >= 1354 AND Prescription: Hydrocodone-Acetaminophen=false AND Number of Affiliated Hospitals >= 4 AND HCPCS: X-ray Exam of Abdomen=true	254	4	98.4%
MI	Number of HCPCS Beneficiaries >= 3038 AND Prescription: Hydrocodone-Acetaminophen=false AND Relative Cost of Procedures <= 0.16 AND HCPCS: CT Thorax with Dye	230	4	98.2%
NJ	NumHCPCSBeneficiaries >= 2706 AND NumHospitals >= 3 AND Prescriptions: Alendronate Sodium=false AND RelativeVolume <= 0.23 AND NumReviews <= 1	338	6	98.2%
TN	Number of HCPCS Beneficiaries >= 2590 AND Prescriptions: Hydrocodone-Acetaminophen=false AND Number of Affiliated Hospitals >= 5 AND Relative Cost of Procedures <= 0.19 AND Prescriptions: Klor-Con 10=false	125	3	97.6%

Table E.2 reports the top five most accurate rules that cover at least 10 providers for Castle Connolly Award=true. Based on the rules from this table, we see that Number of Fellowships and Years of Experience are important, the former appearing in four of the five rules and the latter appearing in all five rules. Further, we observed that three of the five rules contain

attributes related to patient ratings. Thus, attributes that influence Castle Connolly awards differ from state to state, where attributes such as patient reviews or gender have differing influences in differing localities. Illinois presents an interesting rule that says doctors of Internal Medicine with a subspecialty in Pulmonary Disease who have at least one fellowship, participate in PQRS, use EHRs, and see less than 1380 Medicare beneficiaries each year are more likely to receive a Castle Connolly award. We also see an interesting rule in Washington, that says females with at least one fellowship, 20 to 35 years of experience, whose hospital affiliation score is in the top 53%, and who work at organizations with at least 189 employees are more likely to receive a Castle Connolly award.

Table E.2. The top five most accurate features for rules that imply *Castle Connolly Award=true*.

State	Rule	Positive	Negative	Accuracy
TX	Number of Fellowships > 0 AND Number of Organization Members >= 1290 AND Years of Experience > 30 AND Overall Rating >= 25 AND RelativeVolume >= 0.08	25	1	96.1%
IL	Years of Experience >= 25 AND Number of Fellowships > 0 AND EHR=true AND PQRS=true AND Internal Medicine, Pulmonary Disease=true AND Prescription: Levofloxacin=False AND Number of HCPCS Beneficiaries < 1380	15	1	93.7%
OK	Number of Patient Reviews >= 2 AND 86 < Number of Organization Members < 101 AND Years of Experience > 20 AND Medical School Rank >= 39	10	1	90.9%
FL	Years of Experience >= 24 AND Number of Fellowships > 0 AND Number of Patient Reviews >= 3 AND Knowledgeable >= 55 AND 310 < Number of Organization Members < 350 AND Number of Affiliated Hospitals < =1	28	4	87.5%
WA	Gender=Female AND 20 < YearsExp < 35 AND Number of Fellowships > 0 AND Hospital Affiliation Score > 46 AND Number of Organization Members >= 189	19	3	86.3%

References

1. Young A, Chaudhry HJ, Thomas JV, Dugan M. A census of actively licensed physicians in the United States, 2012. Journal of Medical Regulation. 2012;99(2):11-24.
2. Alkhenizan A, Shaw C. Impact of accreditation on the quality of healthcare services: a systematic review of the literature. Annals of Saudi medicine. 2011;31(4):407.
3. Google Developers. Visualization: Map. https://developers.google.com/chart/interactive/docs/gallery/map Accessed Feburary 27, 2016.
4. Creative Commons 3.0 Attribution License. http://creativecommons.org/licenses/by/3.0/ Accessed Feburary 27, 2016.
